# State of the art: refinement of multiple sequence alignments

**DOI:** 10.1186/1471-2105-7-499

**Published:** 2006-11-14

**Authors:** Saikat Chakrabarti, Christopher J Lanczycki, Anna R Panchenko, Teresa M Przytycka, Paul A Thiessen, Stephen H Bryant

**Affiliations:** 1 National Center for Biotechnology Information, National Library of Medicine, National Institutes of Health, Bethesda, MD 20894, USA

## Abstract

**Background:**

Accurate multiple sequence alignments of proteins are very important in computational biology today. Despite the numerous efforts made in this field, all alignment strategies have certain shortcomings resulting in alignments that are not always correct. Refinement of existing alignment can prove to be an intelligent choice considering the increasing importance of high quality alignments in large scale high-throughput analysis.

**Results:**

We provide an extensive comparison of the performance of the alignment refinement algorithms. The accuracy and efficiency of the refinement programs are compared using the 3D structure-based alignments in the BAliBASE benchmark database as well as manually curated high quality alignments from Conserved Domain Database (CDD).

**Conclusion:**

Comparison of performance for refined alignments revealed that despite the absence of dramatic improvements, our refinement method, REFINER, which uses conserved regions as constraints performs better in improving the alignments generated by different alignment algorithms. In most cases REFINER produces a higher-scoring, modestly improved alignment that does not deteriorate the well-conserved regions of the original alignment.

## Background

The reliability and accuracy of many bioinformatics methods such as homolog identification, comparative modeling, phylogenetic analysis and others depend heavily on the quality of multiple sequence alignments. Heuristic approaches such as progressive and iterative methods are generally used to obtain multiple sequence alignments in a computationally efficient manner. In progressive approaches, a multiple alignment is generally built up gradually by aligning the most similar sequences first and successively adding in more distant relatives. A number of alignment programs apply this strategy [1-3] by constructing a global alignment over the entire length of the sequences; they differ mainly in the procedure employed to determine the order of the sequences to be aligned. Iterative algorithms [4,5] generally attempt to improve the overall quality of alignment by employing an objective function and heuristic measures to obtain an optimal alignment. Alternative approaches that utilize a co-operative strategy to integrate complementary algorithms [6,7] and/or incorporate additional biological data [8,9] have also been developed.

Despite the numerous efforts made in this field, each of these strategies has weaknesses that can result in alignments that do not reflect the correct evolutionary relationships. This persistent difficulty of course reflects the fact that aligning multiple sequences is a highly non-trivial task (in both a biological and computational sense) whose accuracy in practice depends largely on the choice of input sequences, the objective function and the heuristics employed. Therefore, the application of an alignment refinement algorithm to an existing or automatically-generated alignment can be helpful for detecting alignment problems. Alignment refinement as a post-processing operation is particularly worthwhile considering the increasing importance of high quality alignments in large scale high-throughput analysis.

Alignment refinement has mainly relied on iterative approaches [4,8,10-12]. Recently Wallace *et al*., [13] studied various iterative schemes and showed that performance of alignment algorithms can be improved by including iteration steps during the progressive alignment. Another refinement program, RASCAL, implemented by Thompson *et al*., [14] uses a knowledge-based strategy to improve alignments where alignment is decomposed into reliable and unreliable regions and only unreliable alignment regions are modified.

Recently we reported a new algorithm, REFINER [15], that refines a multiple sequence alignment by iterative realignment of its individual sequences, using the predetermined conserved core (block) model as a constraint. Realignment of each sequence can correct misalignments between a given sequence and the rest of the profile by shifting the individual aligned blocks on that sequence yet at the same time preserves the family's overall block model (*i.e*., the sequence and structurally conserved regions). The constraint prohibits the insertion of gap characters in the middle of conserved blocks.

In this study we compare the performance of three published alignment refinement algorithms. The accuracy and efficiency of RASCAL [14], the Remove First (RF) method from Wallace *et al*., [13] and REFINER [15] methods were compared using the 3D structure-based alignments from the BAliBASE benchmark database [16] and a collection of manually curated high quality alignments from Conserved Domain Database (CDD) [17]. The quality of the refined alignments was assessed in terms of various scoring functions, by consistency with structure-structure alignments from BAliBASE, and measuring sensitivity in profile-based database searches. As a practical matter, we also report the CPU time required by the three methods. This comparison study reveals that while none of the refinement methods provide dramatic improvements, the REFINER algorithm performs consistently well in conjunction with all alignment-generation algorithms tested. Further, of the three methods studied REFINER best avoids degrading the original alignment's quality.

## Results and discussion

### Improvement of alignment

**Figure 1:**
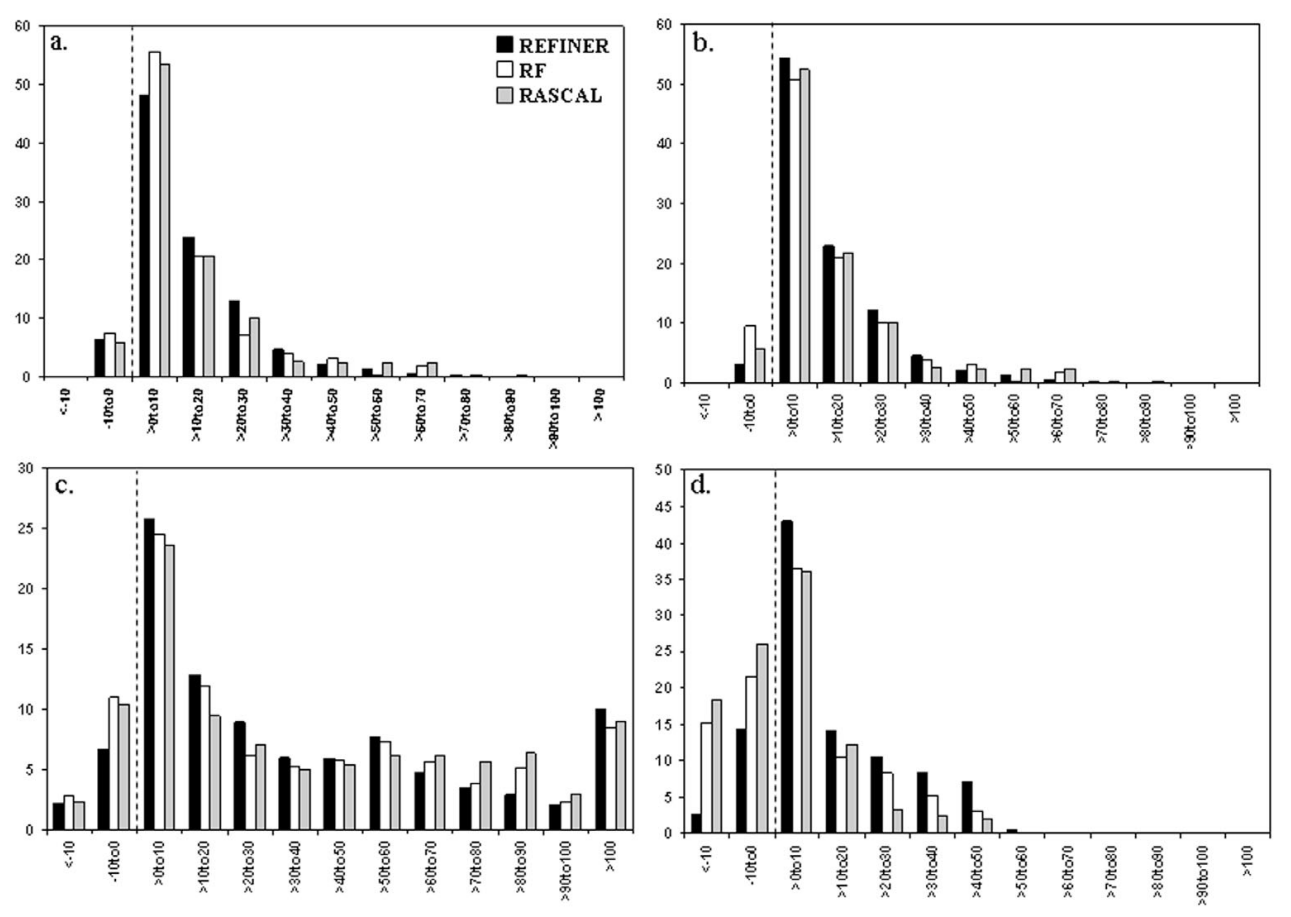
**Improvement of objective scores after refinement (BAliBASE dataset)**. Histograms showing the relative improvement after refinement for four objective scores: a) alignment score b) conservation score (SCORECONS score) c) norMD score and d) information content for the BAliBASE 3.0 alignment dataset are plotted. The X-axis represents bins of relative improvement of the objective score while the Y-axis shows the percentage of alignments. Relative improvement of objective score is measured as the difference between the final scores after application of REFINER, RF and RASCAL method divided by the final score obtained from default alignment program output.

Alignments generated by ClustalW version 1.83 [18], Muscle version 3.52 [19], Dialign version 2.3 [20], FFTNSI from the Mafft package version 5.743 [5,21], ProbCons version 1.09 [22] and TCoffee version 3.93 [7], were refined by three different methods: REFINER [15], RF method [13] and RASCAL [14]. Since each of these refinement methods seeks a multiple alignment with the highest score, we first compared their optimization procedures by calculating scores from the refined alignments using four different objective scores. Figure 1 shows the relative improvement of refinement as measured by alignment score, conservation score [SCORECONS score, [23]], norMD score [24] and information content for the BAliBASE benchmark alignments. The percentage of refined alignments that exhibit an improved score, *i.e*. where the refined alignment has a higher objective score than the original alignments, is found to be highest for REFINER for all objective scores tested. When using alignment score, SCORECONS score, norMD score and information content, respectively, these percentages are 94%, 98%, 90% and 84% for REFINER, 92%, 90%, 86% and 63% for the RF method, and 94%, 94%, 87% and 57% for RASCAL. Notably, these values also reveal that REFINER produces fewer cases in which the objective score of the refined alignments drops.

**Figure 2:**
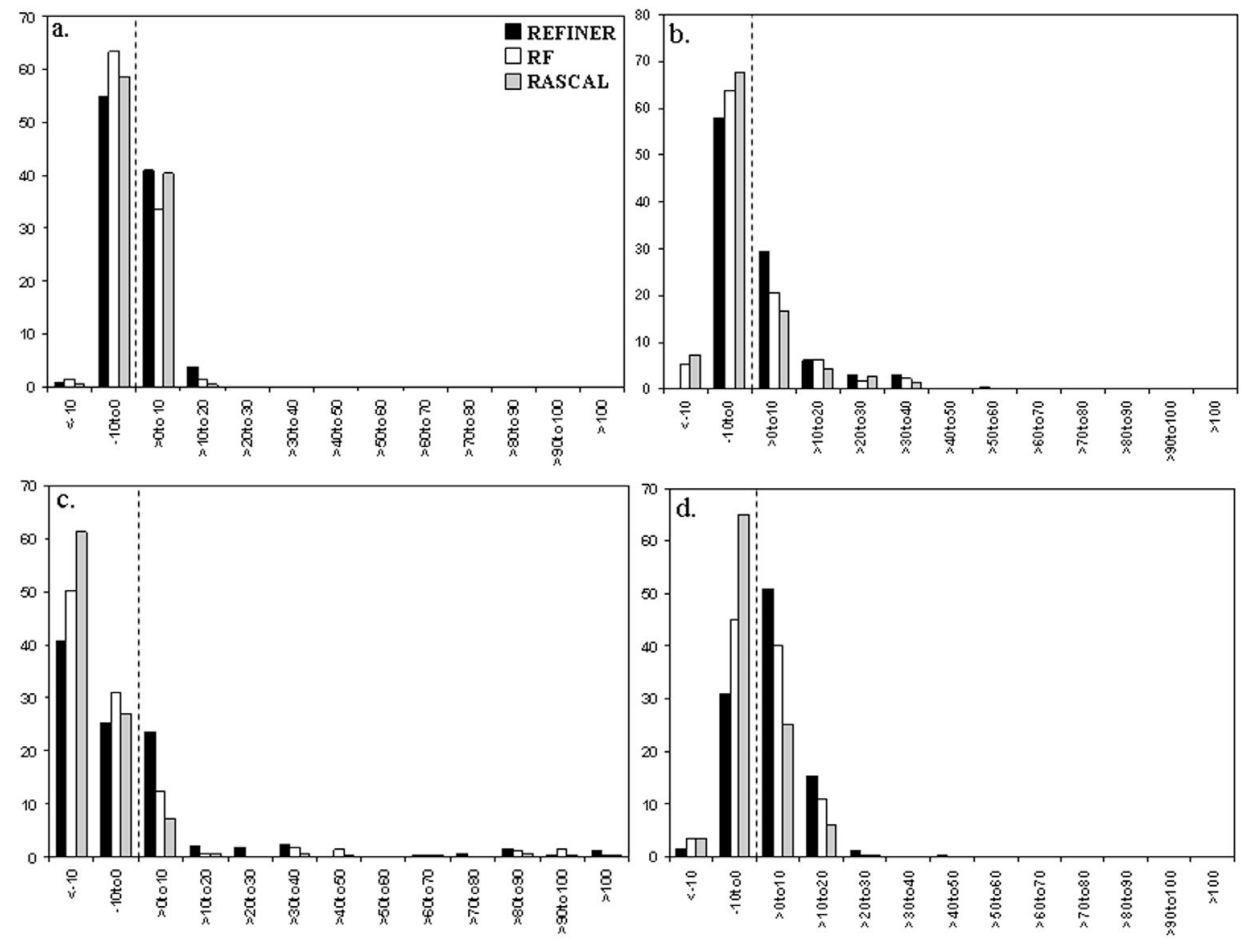
**Improvement of objective scores after refinement (CDD dataset)**. Histograms showing the relative improvement after refinement for four objective scores: a) alignment score b) conservation score (SCORECONS score) c) norMD score and d) information content for the CDD alignment dataset are plotted.

We observe similar results (Figure 2) when REFINER, RF and RASCAL are applied to alignments from the CDD alignment dataset. In this case, refinement algorithms were applied to the original CDD alignments and objective scores are computed pre- and post-refinement. Although the extent of positive improvement (REFINER: 45%, 42%, 34% and 68%; RF: 35%, 31%, 20% and 51% and RASCAL: 41%, 25%, 11% and 32%) is much lower in this dataset, relative to the other methods REFINER performs consistently well for different scoring functions and also results in fewer cases where alignment accuracy actually degrades following refinement.

While REFINER performs well numerically (in terms of the objective score), the biological relevance of the refined alignments should also be considered. In this context the BAliBASE sum-of-pairs (SP) scoring scheme [16] is adopted to evaluate the quality of derived alignments. The improvements in SP score exhibited by the REFINER, RF and RASCAL refinement methods are illustrated in Table 1. The 'Default' columns in Table 1 provide the average SP score for alignments generated by the individual alignment programs used in this benchmarking study, whereas columns under 'RASCAL', 'RF' and REFINER provide the average SP score obtained after applying that refinement method to the corresponding default alignment. It is evident from the table that the REFINER method performs most consistently well in improving the alignments generated by different alignment methods.

**Table 1:** Impact on alignment quality following refinement.

**BAliBASE reference alignments**	**ClustalW**				**Dialign**				**Mafft**			
	
	**Default**	**RASCAL**	**RF**	**REFINER**	**Default**	**RASCAL**	**RF**	**REFINER**	**Default**	**RASCAL**	**RF**	**REFINER**
Reference 1	0.65	0.63	0.66	0.66	0.62	0.65	0.67	0.62	0.70	0.69	0.69	0.71
Reference 2	0.78	0.80	0.80	0.80	0.78	0.80	0.79	0.79	0.83	0.82	0.83	0.82
Reference 3	0.66	0.69	0.67	0.68	0.65	0.64	0.65	0.66	0.76	0.73	0.75	0.77
Reference 4	0.67	0.68	0.66	0.70	0.67	0.71	0.66	0.69	0.75	0.73	0.70	0.77
Reference 5	0.65	0.67	0.66	0.68	0.67	0.65	0.64	0.67	0.76	0.73	0.72	0.76
**Average**	0.682	0.694	0.69	0.704	0.678	0.690	0.682	0.692	0.760	0.740	0.738	0.766
**Increment (%)**	**0**	**1.760**	**1.173**	**3.226**	**0.000**	**1.770**	**0.590**	**2.065**	**0.000**	**-2.632**	**-2.895**	**0.789**

**BAliBASE reference alignments**	**Muscle**				**Probcons**				**T-Coffee**			
	
	**Default**	**RASCAL**	**RF**	**REFINER**	**Default**	**RASCAL**	**RF**	**REFINER**	**Default**	**RASCAL**	**RF**	**REFINER**

Reference 1	0.66	0.66	0.67	0.67	0.72	0.72	0.70	0.73	0.68	0.68	0.69	0.68
Reference 2	0.80	0.81	0.80	0.80	0.83	0.83	0.82	0.82	0.81	0.83	0.82	0.82
Reference 3	0.71	0.71	0.71	0.73	0.76	0.73	0.75	0.77	0.63	0.62	0.62	0.64
Reference 4	0.71	0.72	0.68	0.72	0.77	0.75	0.71	0.77	0.71	0.71	0.70	0.72
Reference 5	0.70	0.71	0.67	0.71	0.76	0.74	0.71	0.75	0.73	0.73	0.69	0.74
**Average**	0.716	0.722	0.706	0.734	0.768	0.754	0.738	0.77	0.704	0.714	0.704	0.720
**Increment (%)**	**0.000**	**0.838**	**-1.397**	**2.514**	**0.000**	**-1.823**	**-3.906**	**0.260**	**0.000**	**1.420**	**0.000**	**2.273**

For comparison, we calculated the correlation coefficients between the improvement of estimated (relative improvement of objective scores) and real alignment accuracy scores (relative improvement of SP score) for the BAliBASE benchmark set. For all scoring methods the correlation coefficient is low (Table 2) for BAliBASE alignments. This could be due to the fact that the real alignment accuracy is estimated from the core regions [16] while objective scores are calculated taking into account the whole alignments. This inconsistency between the correlation of the objective score and SP score in BAliBASE has been reported elsewhere [25]. Nevertheless, on all test sets the objective scores are better correlated to the improvement of real alignment accuracies for REFINER-derived alignments than for RASCAL and RF method refinements.

**Table 2:** Correlation coefficients between the improvements of estimated and real alignment accuracy scores.

	**REFINER**	**RF**	**RASCAL**
**Alignment Score**	0.236	0.188	0.167
**Conservation score**	0.228	0.164	0.162
**norMD score**	0.338	0.308	0.287
**Information content**	0.18	0.15	0.15
**Average**	0.258	0.201	0.191

### Relationship between improvement of alignment accuracy and benchmark difficulty

**Figure 3:**
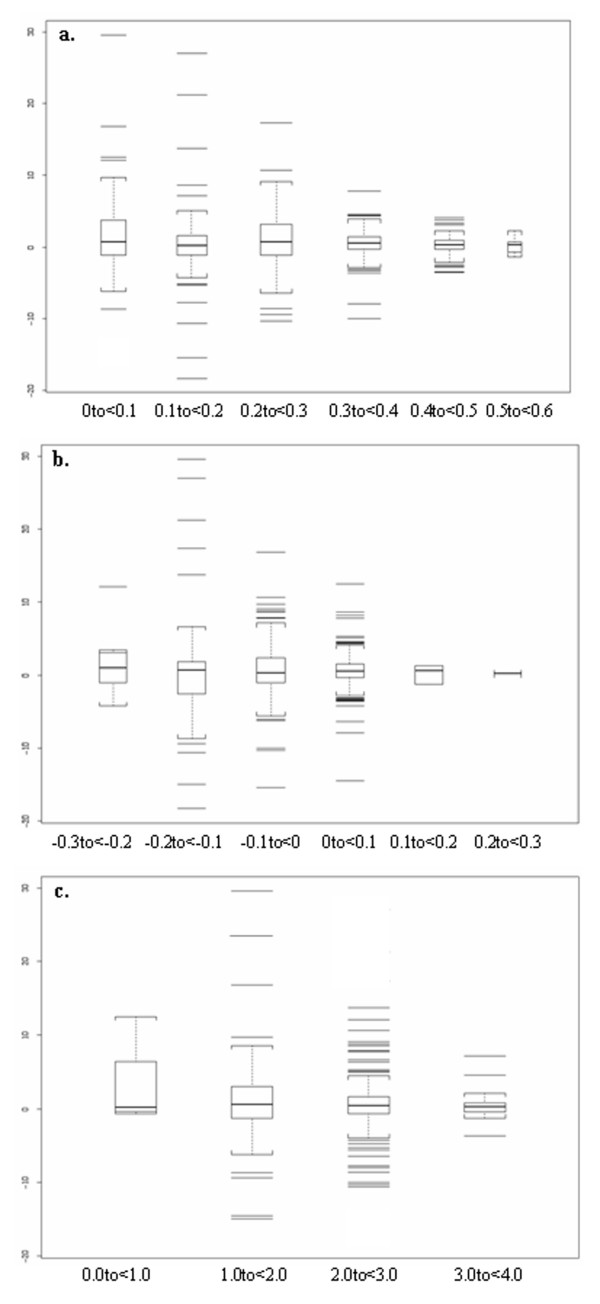
**Relationship between improvement of alignment accuracy and benchmark difficulty**. The relative improvement of the alignment accuracy (Y-axis) calculated as improvement of SP (Sum-of-Pair) score is plotted against the quality of alignment input to REFINER, as measured by three objective scoring functions (X-axis). a) Conservation (SCORECONS) score b) norMD score c) information content. The central line in each box shows the median value, the upper and lower boundaries of individual box show the upper and lower quartiles, and the vertical lines extend to a value 1.5 times the inter quartile range. Outlier values are shown outside the whiskers.

As the quality of an alignment improves, refinement procedures reach a point of diminishing returns. It is therefore useful to identify the circumstances under which refinement is most likely to be helpful. We explored this issue by correlating the improvement of alignment accuracy under refinement (in terms of SP score) and the quality of the initial alignment as measured by its objective score, focusing on the REFINER method. Figure 3 shows the relative improvement of refined alignment accuracy compared to the input alignment's quality as expressed by the SCORECONS score (panel a), norMD score (panel b) and information content (panel c). It can be seen from the figure that alignment refinement has its greatest impact for initial alignments with lower objective scores and it is noteworthy that the alignment accuracy typically gets better upon refinement in these cases. So when the input alignment's score is low (*e.g*. SCORECONS score range [0, 0.1], norMD score range [-0.3, -0.2] and information content range [0, 0.1]) performing alignment refinement is most often beneficial. At higher levels of input alignment quality (*i.e*., higher initial scores), however, it is also encouraging that refinement can provide moderate improvement yet seldom results in significant degradation.

### Comparison of the sensitivity/specificity of the refined alignments

We used the program Hmmsearch from the HMMER 2.3.2 package [26] to perform database searches after converting the refined alignments generated by the REFINER, RF and RASCAL methods to HMM profiles using the program Hmmbuild from the HMMER 2.3.2 package. HMM models derived from the 280 CDD alignments in set_280 (see additional file 1 for list) before and after each of the refinement procedures were used to search the 'non-redundant' database of protein chains (db10185, see Methods for details).

The database search sensitivities at 1% and 5% error rates are given in Table 3. The sensitivities of sequence profiles/HMMs have increased only when employing REFINER, and the database search sensitivities fall slightly for the other two refinement methods. Admittedly the overall improvement in sensitivity is not dramatic but it does imply that in this scenario the REFINER algorithm produces better refined alignments.

**Table 3:** Sensitivity values estimated from the ROC curves at 1% and 5% error rates (fraction of false positives)

**Search method**	**Error rate**	**Original**	**After_REFINER**	**After_RF**	**After_RASCAL**
**HMMER**	**1% error rate**	0.47	0.48	0.46	0.45
	**5% error rate**	0.54	0.56	0.54	0.54

### Comparison of average run times for refinement

Finally, we compare the average computation time required for each of the refinement algorithms. The CPU time cost is an important practical factor that influences the utility of an algorithm to a great extent. Table 4 shows the average CPU time for refinement of five reference benchmark sets from BAliBASE 3.0 for each of six alignment-generation programs. The 'Default' column displays the time spent generating the alignment used as input to each refinement program. RASCAL proves to be the fastest refinement method while RF method seems to be very expensive in comparison. REFINER's runtime characteristics are similar to those of RASCAL for all five BAliBASE references.

**Table 4:** Comparison of average run time (in seconds) for the BAliBASE 3.0 benchmark dataset.

**BAliBASE reference alignments**	**ClustalW**				**Dialign**				**Mafft**			
	
	**Default**	**RASCAL**	**RF**	**REFINER**	**Default**	**RASCAL**	**RF**	**REFINER**	**Default**	**RASCAL**	**RF**	**REFINER**
Reference 1	10.58	10.34	51.06	1.93	22.21	10.00	38.46	1.65	26.03	10.33	53.55	1.67
Reference 2	64.93	12.62	206.58	26.67	175.67	12.61	179.62	18.97	123.58	11.61	129.75	18.85
Reference 3	98.81	25.84	495.25	35.98	304.23	24.84	397.11	35.60	241.69	21.69	237.96	32.35
Reference 4	49.79	23.04	370.85	13.95	496.04	24.08	607.02	11.18	222.70	22.08	271.87	8.91
Reference 5	35.66	13.23	327.00	15.19	201.33	12.00	300.00	12.93	103.00	10.00	209.00	13.45
**Average**	**51.95**	**17.01**	**290.15**	**18.74**	**239.90**	**16.71**	**304.44**	**16.07**	**143.40**	**15.14**	**180.43**	**15.05**

**BAliBASE reference alignments**	**Muscle**				**Probcons**				**T-Coffee**			
	
	**Default**	**RASCAL**	**RF**	**REFINER**	**Default**	**RASCAL**	**RF**	**REFINER**	**Default**	**RASCAL**	**RF**	**REFINER**

Reference 1	23.21	10.00	49.83	2.09	29.34	10.54	38.67	2.37	20.54	10.62	36.79	1.73
Reference 2	116.66	12.20	124.50	16.10	167.12	12.25	170.50	13.47	160.74	21.23	166.29	18.34
Reference 3	267.28	24.84	261.86	36.76	289.49	20.84	283.72	31.38	284.11	21.78	265.29	38.84
Reference 4	267.70	24.58	272.55	11.00	362.29	22.08	443.12	11.26	403.40	12.08	415.00	7.57
Reference 5	111.00	8.00	215.00	20.41	203.00	8.00	206.00	15.64	188.00	10.00	232.00	14.03
**Average**	**157.17**	**15.92**	**184.75**	**17.27**	**210.25**	**14.74**	**228.40**	**14.82**	**211.36**	**15.14**	**223.07**	**16.10**

Although the computational complexity may be similar for RF and REFINER due to their similar approaches to the problem, implementation differences appear to account for the disparity in speed between the two methods. Specifically, RF is a Perl script which performs multiple system calls within its innermost iteration loop, including invocations of the program(s) Muscle and/or ClustalW to perform sequence realignment operations. In contrast REFINER is a C++ binary that has no nested system calls and includes a fast dynamic programming engine to do sequence realignment operations.

## Conclusion

The alignment of multiple sequences is a very important task and still remains a challenging problem. Acknowledging the difficulty of that challenge, an alternate approach to the underlying problem has been examined here: augmenting alignment-generation procedures with a separate alignment-refinement algorithm capable of repairing those errors that remain. Iterative refinement algorithms generally attempt to improve the overall quality of alignment by employing objective functions and heuristics to obtain an optimal alignment. Most iterative refinement methods face the challenge of how to escape from sub-optimal alignments. Therefore, the main differences among the existing methods lie in the effective definition of an objective function and intelligent design of the method's heuristics.

In this study we conducted an extensive comparison of the performances of three different alignment refinement algorithms. The accuracy and efficiency of the refinement programs such as, RASCAL [14], RF method [13] and REFINER [15] were compared using the 3D structure-based alignments from the BAliBASE benchmark database as well as a diverse set of manually curated high quality alignments from the Conserved Domain Database. A comparison in terms of different objective scoring functions found better performance for alignments refined by REFINER rather than the RF or RASCAL methods. The biological relevance of the refined alignments was also assessed, where we adopted the BAliBASE sum-of-pair (SP) scoring scheme to evaluate the refined alignments' quality. Though none of the methods displayed dramatic improvements, REFINER performed consistently well for alignments generated by six different alignment algorithms. Correlation analysis between improvements in the predicted accuracy (objective score) and the real accuracy (SP score) also suggested better overall performance by REFINER algorithm.

Further, we tried to identify the range of initial alignment quality in which REFINER is most successful at improving the alignment. High-quality input alignments are difficult for refinement algorithms to improve without also making additional deleterious modifications. Because the good input alignments also tend towards higher objective scores, for these purposes the input alignment's objective score is viewed in a general sense a proxy for the difficulty that alignment presents to a refinement algorithm. The impacts of refinement by the REFINER algorithm are very prominent in the lower ranges of initial alignment quality where REFINER provided significant improvements. For higher quality input alignments (*i.e*., higher ranges of the input's objective score) although REFINER still found improvements, the impact of refinement is reduced. This might indicate that those alignments were already been optimized and therefore were less prone to changes.

We have also described a way to validate the quality of a refined alignment by examining the performance of its sequence profile in homology searches. This validation test provides a useful quality control in the typical situation where one does not have a reference alignment. In addition, it demonstrated that the sensitivity of sequence profiles/HMMs increased when employing the REFINER method but fell slightly for the other two refinement methods studied.

Since the REFINER method was designed as an alignment refinement tool, extensive benchmarking, validation and comparison of its performance is vital. Therefore, we conducted comparison tests on large benchmark data sets and found that REFINER on average provided moderately better performance in terms of improving the quality of an input alignment. However, we have also shown that significant improvements are possible, particularly for initial alignments with lower values of one of the various common objective functions. Obtaining such improvements manually or by re-running another automated alignment generation algorithms is both uncertain and time-consuming. Therefore, as a practical matter, refinement methods such as REFINER do appear well worth the time spent on their application to alignments of interest.

Our previous study [15] established the concept that realignment of each sequence can correct misalignments between a given sequence and the rest of the profile and at the same time preserves the family's overall block model. In contrast, the current manuscript describes a comparison of three different methods available for the refinement of multiple sequence alignments using a standard benchmark dataset (BAliBASE 3.0). The performance of the refinement methods is compared in terms of profile sensitivities for homolog retrieval and CPU time usage. Furthermore, we analyzed how different strategies for using refinement programs are appropriate depending on the quality of the input alignment (*i.e*., its difficulty). We are not aware of another analysis like the one presented here, and believe that it will be helpful to researchers in the sequence analysis field when attempting to decide if their alignment tasks can benefit from the use of one or more refinement programs.

## Methods

### Benchmark dataset

We used the BAliBASE 3.0 [16] alignment benchmark set and a subset of the Conserved Domain Database (CDD) [17] to validate the performance of different alignment refinement methods. The BAliBASE benchmark set contains 386 reference alignments, whereas our CDD dataset collects 280 manually curated 'root alignments' from CDD version 2.03. (CDD organizes related curated alignment models into hierarchies; a root alignment corresponds to the top-level alignment in a CDD hierarchy). To compare the sensitivity of profiles constructed from refined alignments, we used these 280 root alignments (set_280, see additional file 1 for list).

### Overview of the refinement algorithms

#### REFINER

The REFINER algorithm refines an existing alignment by systematically realigning each sequence to the profile constructed from the remaining sequences in the family. The algorithm performs one or more iterations of refinement; each iteration contains a phase of 'block shifting' followed by a 'block editing' phase. The refinement is constrained by the block model defined for a given alignment, where a block is a region containing no gaps on any sequence; a block is specified simply by a start position and residue length. The order in which the sequences are realigned (using a fast block-based dynamic programming algorithm) is randomized to avoid bias and make the use of multiple iterations more effective. Convergence is declared when no further improvement of overall alignment score is observed or all iterations have been performed. A detailed description of the algorithm can be found in Chakrabarti *et al*., 2006 [15].

#### RF method

The Remove First (RF) scheme from Wallace *et al*., [13] is similar to REFINER but it does not use constraints imposed by the block structures. In each iteration step of the RF method a sequence is realigned to the remaining alignment and if the resulting alignment is better, it is kept and used as input for the next iteration. The iteration cycle is terminated if the alignment score converges, or upon completing 2*N*^2 ^iterations, where *N *is the number of sequences. Two different programs were used to align sequences in this protocol; ClustalW [18], which maximizes the Average Score, and Muscle [19], which maximizes the logarithmic expectation (LE) score. In this study we compared the quality of alignments after refinement by RF method using LE scoring scheme.

#### RASCAL

The refinement program RASCAL [14] uses a different algorithmic approach than RF and REFINER. RASCAL first clusters multiple sequence alignments into potential functional subfamilies and identifies well-aligned, reliable regions in each subfamily. RASCAL then performs a single realignment of each badly aligned region using an algorithm similar to that implemented in ClustalW [18].

### Alignment programs used to align BAliBASE dataset

Six multiple alignment programs were used to generate alignments as inputs for the refinement algorithms. These are: ClustalW version 1.83 [18], Muscle version 3.52 [19], Dialign version 2.3 [20], FFTNSI from the Mafft package version 5.743 [5,21], ProbCons version 1.09 [22] and TCoffee version 3.93 [7]. The default settings of the parameters were used for each alignment program.

### Quality assessment

When using the BAliBASE benchmark, we used the bali_score [16] program to assess the accuracy of each test case. The Sum-of-Pair (SP) scoring scheme is employed to test the accuracy of refined alignments. The SP score is the ratio of the number of correctly aligned pairs of core block positions in the test alignment to the number of aligned pairs in the reference alignment.

In the case of CDD alignments we evaluate the refined alignments by calculating the objective scores SCORECONS [23] and norMD [24], both having been previously suggested for the analysis of alignment quality. In addition, we calculated the alignment score and information content for each alignment as a measure of quality. The alignment score is measured as the sum of Position Specific Scoring Matrix (PSSM) scores over all aligned positions of an alignment. Information content was calculated based on counting the number of different amino acid types per aligned column and comparing with the number expected based on standard Robinson & Robinson [27] amino acid background frequencies.

Another way to validate alignment refinement methods is to examine the performance of a refined alignment in homology-based database searches. To compare the database search sensitivity of the profiles or Hidden Markov Models (HMMs) computed from alignments before and after the refinement procedure, we first constructed a list of true positives for the conserved domain families from set_280. True positives here were defined as those proteins/domains which were structurally similar, as defined by the VAST algorithm [28,29], to the representative structure of CDD alignments. First, for each CDD alignment we chose a representative structure so that the CDD footprint on this structure and the corresponding structural domain/chain boundaries (domain definitions from MMDB structure database have been used [30]) are consistent to a degree of 80% mutual overlap. By CDD footprint we mean the region on a structure between the first and the last residues aligned in CDD. For CDD alignments that have a corresponding MMDB structural domain, the VAST structure neighbors of an MMDB domain/chain are retrieved from the non-redundant set of MMDB chains. This set of 10185 chains (db10185) was constructed by single-linkage clustering, based on BLAST *E*-values of 10^-80 ^or less, from all the entries in the MMDB structure database [30].

We used HMMER [26] to test the ability of the refined sequence profiles to find the corresponding VAST neighbors in the db10185 dataset of structural chains. The sensitivity-specificity analysis was performed by calculating Receiver Operating Characteristic (ROC) curves and ROC statistics. For a given protein family one can calculate the fraction of detected true positives and false positives at each similarity measure cutoff (E-value for HMMER). Sensitivity here is defined as the number of true positives detected divided by the overall number of true positives in the database. The fraction of false positives is calculated as the ratio between the number of false positives found and the overall number of false positives in the database. To compare profile sensitivity before and after the refinement we made measurements at 1% and 5% false positive error rates.

## Abbreviations

CDD Conserved Domain Database

SP Sum-of-Pairs

HMM Hidden Markov Models

PSSM Position Specific Scoring Matrix

CPU Central Processing Unit

## Authors' contributions

S.C. had carried out the benchmarking, was involved in the development and validation of the algorithm and has written the first draft of the manuscript. C.J.L and P.A.T were involved in development of the program. T.M.P and S.H.B were involved in discussion and critical reading of the manuscript. All authors were involved in the design of the algorithm. S.C, C.J.L, A.R.P, T.M.P and S.H.B participated in discussions and writing the final version of the paper.
