# State of the art: refinement of multiple sequence alignments

**DOI:** 10.1186/1471-2105-11-3

**Published:** 2010-01-04

**Authors:** Saikat Chakrabarti, Christopher J Lanczycki, Anna R Panchenko, Teresa M Przytycka, Paul A Thiessen, Stephen H Bryant

**Affiliations:** 1 National Center for Biotechnology Information, National Library of Medicine, National Institutes of Health, Bethesda, MD 20894, USA

## Abstract

Correction to Chakrabarti S, Lanczycki CJ, Panchenko AR, Przytycka TM, Thiessen PA and Bryant SH: State of the art: refinement of multiple sequence alignments. BMC Bioinformatics 2006, 7:499.

## Correction

After publication of [1] we have noticed an error in our manuscript. We have realized that there were some numbers incorrectly inserted within the Table 1 of the manuscript [1]. Corrected values are now provided In Table 1, for six of the numbers that were entered incorrectly: the SP scores for References 1, 4 and 5 under Muscle reference alignment (REFINER refinement alignment) and, SP scores for References 2, 3 and 5 under T-Coffee reference alignment (Default refinement alignment). However, this does not affect our original interpretation of the data presented in our original publication in any way. We regret any inconvenience that this inaccuracy might have caused.

**Table 1:** Impact on alignment quality following refinement.

BAliBASE reference alignments	ClustalW				Dialign				Mafft			
	Default	RASCAL	RF	REFINER	Default	RASCAL	RF	REFINER	Default	RASCAL	RF	REFINER
Reference 1	0.65	0.63	0.66	0.66	0.62	0.65	0.67	0.62	0.70	0.69	0.69	0.71
Reference 2	0.78	0.80	0.80	0.80	0.78	0.80	0.79	0.79	0.83	0.82	0.83	0.82
Reference 3	0.66	0.69	0.67	0.68	0.65	0.64	0.65	0.66	0.76	0.73	0.75	0.77
Reference 4	0.67	0.68	0.66	0.70	0.67	0.71	0.66	0.69	0.75	0.73	0.70	0.77
Reference 5	0.65	0.67	0.66	0.68	0.67	0.65	0.64	0.67	0.76	0.73	0.72	0.76
**Average**	0.682	0.694	0.69	0.704	0.678	0.690	0.682	0.692	0.760	0.740	0.738	0.766
**Increment (%)**	**0**	**1.760**	**1.173**	**3.226**	**0.000**	**1.770**	**0.590**	**2.065**	**0.000**	**-2.632**	**-2.895**	**0.789**

**BAliBASE reference alignments**	**Muscle**				**Probcons**				**T-Coffee**			
	**Default**	**RASCAL**	**RF**	**REFINER**	**Default**	**RASCAL**	**RF**	**REFINER**	**Default**	**RASCAL**	**RF**	**REFINER**

Reference 1	0.66	0.66	0.67	0.68	0.72	0.72	0.70	0.73	0.68	0.68	0.69	0.68
Reference 2	0.80	0.81	0.80	0.80	0.83	0.83	0.82	0.82	0.8	0.83	0.82	0.82
Reference 3	0.71	0.71	0.71	0.73	0.76	0.73	0.75	0.77	0.61	0.62	0.62	0.64
Reference 4	0.71	0.72	0.68	0.74	0.77	0.75	0.71	0.77	0.71	0.71	0.70	0.72
Reference 5	0.70	0.71	0.67	0.72	0.76	0.74	0.71	0.75	0.72	0.73	0.69	0.74
**Average**	0.716	0.722	0.706	0.734	0.768	0.754	0.738	0.77	0.704	0.714	0.704	0.720
**Increment (%)**	**0.000**	**0.838**	**-1.397**	**2.514**	**0.000**	**-1.823**	**-3.906**	**0.260**	**0.000**	**1.420**	**0.000**	**2.273**
